# Dr. Abercrombie on a Peculiar Delirium

**Published:** 1830-01-01

**Authors:** 


XXIV.

### On a peculiar Delirium, unaccompanied after Death with the usual Appearances of Inflammation of the Brain.

In the first edition of his work Dr. Abercrombie described a peculiar affection of the brain under the head of, "*A dangerous Modification of Meningitis which shows only increased vascularity.*" The following is the graphic description of the disease originally furnished by our author.

"Another important modification of the disease occurs in an insidious and highly dangerous affection, which I think has been little attended to by writers on the diseases of the brain. It is apt to be mistaken for mania, or, in females, for a modification of hysteria; and in this manner the dangerous nature of it has sometimes been overlooked, until it proved rapidly and unexpectedly fatal. It sometimes commences with depression of spirits, which after a short time passes off very suddenly, and is at once succeeded by an unusual degree of cheerfulness, rapidly followed by maniacal excitement. In other cases, these preliminary stages are less remarkable; the affection, when it first excites attention, being in its more confirmed form. This is in general distinguished by remarkable quickness of manner, rapid incessant talking, and rambling from one subject to another, with obstinate watchfulness, and a small frequent pulse. Sometimes there is hallucination or conception of persons or things which are not present, but in others this is entirely wanting. The progress of the affection is generally rapid; in some cases, it passes into convulsion and coma; but in general it is fatal by a sudden sinking of the vital powers, supervening upon the high excitement, without coma. The principal morbid appearance is a highly vascular state of the pia mater, sometimes with very slight effusion betwixt it and the arachnoid. The disease is one of extreme danger, and does not in general admit of very active treatment. General bleeding is not borne well, and the treatment must in general be confined to topical bleeding with purgatives, antimonials, and the powerful application of cold to the head. The affection is most common in females of a delicate irritable habit, but also occurs in males, especially in those who have been addicted to intemperance. I have however seen it in one case, in a gentleman between 40 and 50, of stout make and very temperate habits. The cause of death is obscure; it seems in general to be a sudden sinking of the vital powers, supervening upon the high excitement without any of the actual results of inflammation."

We think that most of our readers will be struck with a general resemblance between the affection thus described, and that class of maniacal disorders or hallucinations which attacks hysterical or debilitated women, and intemperate men. It is a class of affections characterised rather by the excitement of the nervous than the vascular systems, frequently benefited by what tends to lull the irritability of the former, almost always injured by active measures directed against the latter. The pervigilium, quickness of manner, incessant talking, and small frequent pulse are symptoms that with little alteration or addition would fit the delirium tremens, as well as if they were made for it. They bear too a striking resemblance to the characters of some of those puerperal affections so well described of late by Dr. Gooch. But to revert to our author, it seems that since the first edition of his work he has modified his opinions on the treatment of the disease, and now recommends more stimulating measures. On a question of such practical, we may really say vital importance let Dr. A. speak for himself.

"The above remarks on this highly dangerous and interesting affection, I leave as they stood in the first edition of this work. Since that time I have seen several examples of it, and have been induced to adopt a different mode of treatment, which seems to promise some interesting results. Without at present venturing upon any general conclusions, I shall merely submit the following case.

"Case XVIII.—A lady, aged about 38, was recovering from her eleventh accouchement, when, at the end of a fortnight, she became affected with a deep-seated hard swelling in the right side of the pelvis, which was tender to the touch, and was accompanied by a considerable degree of fever. After repeated topical bleeding and other remedies, the febrile state subsided, the swelling lost its tenderness, and seemed to be gradually diminishing in size; but its progress was very slow, and after three or four weeks, she was still confined to bed, and suffering a good deal of uneasiness; her pulse was now calm, but she was considerably reduced in strength. At this time, she became, one day, alarmed and agitated by some family occurrence, and immediately began to talk wildly and incoherently, and after a restless night was found next day in a state of the highest excitement, talking incessantly, screaming and struggling, with a wild expression of countenance, and a small rapid pulse. She was treated by topical bleeding, laxatives, cold applications to the head, &c., but with little or no benefit; and on visiting her on the following day, I found her sitting up in bed, with a look of extreme wildness, both her hands in constant motion, talking incessantly and wildly; and I learnt that she had not ceased talking for one instant for the last twelve hours. Her pulse was now rapid and feeble, and her countenance expressive of exhaustion. In consultation with a highly intelligent friend who had charge of the case, I mentioned my experience of the fatal nature of the affection, and proposed to make trial of treatment by stimulants. A glass of wine was accordingly given, with evident abatement of the symptoms; and it was ordered to be repeated every hour. At the end of the fourth hour, she was perfectly composed and rational, her pulse about 90 and of good strength; and from this time there was no return of the symptoms. The tumour in the right side increased in size, suppurated, was opened, and healed favourably. From this time she continued in perfect health, and has since passed through another accouchement in the most favourable manner.

"This case I have given as another example of this interesting affection. I have employed the same mode of treatment, with similar benefit in several other cases, both of males and females. The chief difficulty is in deciding upon the particular cases to which the stimulating treatment is applicable. They appear to be those in which the excitement is accompanied by a small and rapid pulse, and an expression of paleness and exhaustion. When these characters are present, however violent the excitement may be, I have not been deterred from the practice, and in a considerable number of instances have found much reason to be satisfied with it. I have tried it, but without the same benefit, in some of the common cases of insanity, accompanied by paleness and bodily weakness, but with a natural pulse. When there is frequent and strong pulse, with flushing and other marks of increased vascular action, it would of course be injurious."

The above results of much experience and acute observation are well worthy the attention of our readers.

